# LEACH Protocol Optimization Based on Weighting Strategy and the Improved Ant Colony Algorithm

**DOI:** 10.3389/fnbot.2022.840332

**Published:** 2022-03-18

**Authors:** Xuezhen Cheng, Chuannuo Xu, Xiaoqing Liu, Jiming Li, Junming Zhang

**Affiliations:** ^1^College of Electrical Engineering and Automation, Shandong University of Science and Technology, Qingdao, China; ^2^Shandong Senter Electronic Co, Zibo, China; ^3^College of Energy and Mining Engineering, Shandong University of Science and Technology, Qingdao, China

**Keywords:** optimal combination weighting, improved ant colony optimization, path superiority, LEACH optimization, routing protocol

## Abstract

This article aims to address problems in the current clustering process of low-energy adaptive clustering hierarchy (LEACH) in the wireless sensor networks, such as strong randomness and local optimum in the path optimization. This article proposes an optimal combined weighting (OCW) and improved ant colony optimization (IACO) algorithm for the LEACH protocol optimization. First, cluster head nodes are updated *via* a dynamic replacement mechanism of the whole network cluster head nodes to reduce the network energy consumption. In order to improve the quality of the selected cluster head nodes, this article proposes the OCW method to dynamically change the weight according to the importance of the cluster head node in different regions, in accordance with the three impact factors of the node residual energy, density, and distance between the node and the sink node in different regions. Second, the network is partitioned and the transmission path among the clusters can be optimized by the transfer probability in IACO with combined local and global pheromone update mechanism. The efficacy of the proposed LEACH protocol optimization method has been verified with MATLAB simulation experiments.

## Introduction

**Figure 1:**
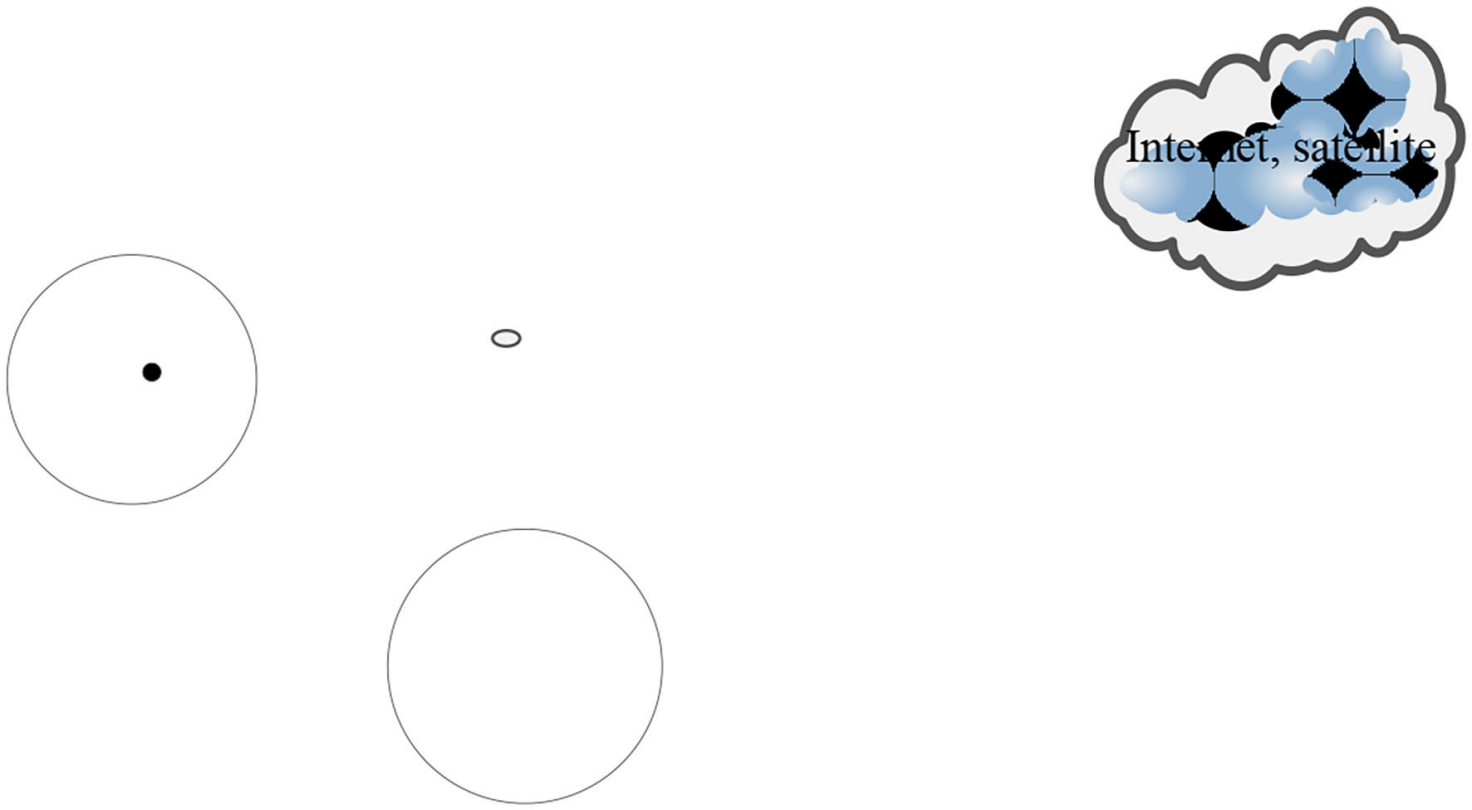
Network structure diagram of LEACH protocol.

It is known that wireless sensor networks (WSNs) are composed of many spatially distributed sensor nodes with limited energy (Efe et al., 2013), whereas the sensor nodes are usually powered by light batteries. Frequent charging or battery replacement of the sensor nodes would cause inconvenience to maintenance; hence, balancing the energy consumption of sensor nodes and prolonging the network lifetime are the two most important indicators to evaluate the performance of the WSNs (Tripathi et al., 2013; Mukherjee et al., 2018). The energy loss of the network can directly affect the performance and life of the network, which should be delicately dealt with to keep the low energy loss of the network in the communication process (Yan et al., 2018; Mohar et al., 2020; Lv et al., 2021). Many routing protocols can be used in WSNs, where hierarchical routing protocols are the most widely adopted. The typical hierarchical routing protocols include low-energy adaptive clustering hierarchy (LEACH), power-efficient gathering in sensor information systems (PEGASIS), threshold-sensitive energy-efficient sensor network (TEEN) protocol, and hybrid energy-efficient distributed (HEED) clustering approach, which can gather the nodes into clusters to form a specific hierarchy (Galkin, 2018). Particularly, the typical LEACH protocol was proposed by Heinzelman (2000) and adopted the “wheel” cycle mode for the first time, which is widely applied due to its low power consumption, node equality, and self-clustering adaptation. In such a protocol, each sensor node contains a clustering algorithm and a data transmission algorithm among nodes (Chen et al., 2015), where the clustering algorithm can randomly change the cluster head nodes by comparing the size of the random number and threshold. After deployment, all sensor nodes self-organize to form different clusters. Generally, each cluster contains a cluster head node and multiple sensor member nodes. The nodes of the specific cluster can only hop one step inside the cluster to the cluster head. The cluster head node of each cluster will also hop one step to transmit the received information to the sink node to complete an iteration circle. The structure of the LEACH protocol is illustrated in Figure 1.

In the LEACH protocol, the energy of the sensor nodes is evenly distributed, and the network duration can be prolonged by balancing the energy consumption of the sensor nodes (Wang et al., 2018). However, the random selection of the cluster head nodes in the protocol could lead to uneven distribution of the cluster head nodes, which is prone to a large or small number of member nodes in the cluster (Marappan and Rodrigues, 2016). Each cluster head node and the base station adopt a single-hop transmission mode, which could easily cause premature death of the remote cluster head, poor expansibility, and uneven energy consumption phenomena in the network. The replacement mechanism of clusters using wheels will speed up the replacement frequency of the cluster head, which could result in more times of the information transmission in the network and shortened network life (Rahman et al., 2013; Jameii and Maadani, 2016). Then, the LEACH protocol has been improved with the fractional lion (FLION) algorithm to generate the optimal route (Sirdeshpande and Udupi, 2017), whereas the fractional derivative is introduced to detect the neighbor solution, and the forward link algorithm is also used to select channels to improve the network survival duration. However, the distribution density of the nodes is not considered in the fitness function. Lalwani et al. (2018) used the harmony search algorithm (HSA) to determine the optimal routing, where the fitness function is designed considering the node density, energy, and distance factor, by choosing the smallest distance nodes for data transmission so as to achieve the energy consumption reduction of the nodes. However, when the cluster contains non-local nodes, the network performance will be affected. Ning et al. (2017), Ezhilarasi (2019) adopted an improved particle swarm optimization (PSO) to optimize the clusters of the WSN process. However, the selection rules of the cluster head node are relatively complex, and the cluster scale rapid update frequency consumes high energy due to fast convergence. At the same time, local optimum would easily occur. Hence, certain algorithms are proposed to tackle such problems, where the cuckoo search algorithm (CSA) (Huang and Hua, 2020) and the fruit fly algorithm (FFA) (Dai et al., 2020) are developed to optimize the cluster and routing protocol and to determine the transmission path according to the distance, energy, and trust value from the node to the base station such factors. However, the generalization capability of the surviving nodes during communication has not been considered (Maheshwari et al., 2021).

In recent years, some improved heuristic intelligent algorithms have become research hotspots. For instance, the ant colony optimization (ACO) algorithm has been extensively studied and applied since it can assist to select the optimal path for the fused data transmission and the network life cycle extension (Ding, 2020). Experts and scholars have explored the ACO algorithms to reduce the energy consumption of the nodes. An ACO multipath routing protocol has been proposed based on the angle factor and entropy to optimize the cluster head nodes with high reliability in the process of data transmission (Hou et al., 2017; Liu and Li, 2018). To tackle the energy consumption balance problem, Zou and Qian (2019) proposed improved ACO (IACO) with a sensor node transfer function and pheromone updating routing rules for optimal WSN routing but with little consideration of the impact factors. Nayyar and Singh (2020) proposed an energy-saving multipath routing protocol based on the ACO algorithm for dynamic WSNs, in which the optimal path for the adjacent nodes is designed, but the scalability is poor. Therefore, it is of great significance to investigate the method to improve the transmission performance of the WSNs and select the optimal data transmission path quickly and effectively.

This article aims to propose an IACO algorithm for the LEACH protocol optimization. The specific sections are as follows: the first section is the improvement of the clustering algorithm based on optimal combination weighting. Furthermore, the designed transmission path between clusters based on the IACO algorithm is proposed in section Improved Clustering Algorithm Based on OCW. Simulation experiments for the proposed LEACH protocol optimization verification are described in section Energy Consumption Analysis of the Sensor Node. The Conclusion is given in section Optimization of the Transmission Path Between Clusters Based on IACO. Table 1 compares the energy-saving, cluster establishment time, uniform distribution of cluster head nodes, and path selection of the representative typical routing protocols.

**Table 1:** WSNs cluster routing protocol performance comparison.

**Protocol**	**Energy saving performance**	**Cluster creation time**	**Uniform distribution of cluster head nodes**	**Path selection**
LEACH	Poor	Quick	Difference	Single jump
TEEN	Good	Faster	Difference	Single jump
PEGASIS	Better	Slow	–	More jumps
HEED	Better	Slower	Good	More jumps
EEUC	Good	Medium	Medium	More jumps

## Improved Clustering Algorithm Based on OCW

### The Update Mechanism of the Cluster Head Node

In the LEACH, cluster head nodes are replaced at each round, which would increase the network power consumption. Therefore, the cluster head nodes are updated within the existed clusters for the whole network clusters. The node whose energy is lower than the average energy *E*_*a*_ in the cluster loses the qualification to be the cluster head node, whereas the node with the highest remaining energy in the cluster is defined as the cluster head node of the next round, and the average energy *E*_*a*_ in the cluster is defined as,


(1) $$\begin{array}{l} E_{a}=\sum \frac{E_{i}}{N_{alive}} \end{array}$$

where *E*_*i*_ is the remaining energy of the surviving node *i* in the cluster and *N*_*alive*_ is the total number of the surviving nodes. If the energy of the current cluster head nodes is lower than this value, WSNs update the cluster size.

The average energy *E*_*all*_ of all the cluster head nodes in the whole network is calculated as,


(2) $$\begin{array}{l} E_{all}=\sum \frac{E_{r}}{B} \end{array}$$

where *E*_*r*_ and *B* are the residual energy and the total number of the cluster head nodes.

### The Threshold of the Cluster Head Node Selection

#### Improved Threshold for Cluster Head Node Selection

The improved LEACH protocol has the same rules as cluster head selection in LEACH protocol, while the selection threshold is defined as *T*_*imp*_(*n*), written as,


(3) $$T_{imp}\left(n\right)=\{\begin{array}{c} \omega_{1}\frac{E_{i}}{E_{0}}+\omega_{2}\frac{E_{alive}}{N_{alive}}+\omega_{3}\frac{d_{\max}-d_{is}}{d_{\max}-d_{\min}}\;,\;n\in G\\ 0\;,\;n\notin G \end{array}$$

$$\begin{array}{l} R=\sqrt{\frac{S}{\pi Np}}\\ l_{alive}=\left\{S_{j}|d_{ij}\leq R,S_{j}\in S\right\} \end{array}$$

where *E*_0_ is the initial energy of the survived node, *l*_*alive*_ is the number of the existing neighboring nodes, *d*_max_ is the distance from node *i* to the sink node, *d*_max_ and *d*_min_ are the farthest and closest distance from the surviving node to the sink node, respectively, and {ω_1_, ω_2_, ω_3_} are the weights of the node energy consumption, density, and distance, respectively.

In the improved threshold, the probability of being selected as the cluster head node is related to the energy, density, and distance from the sink node. As the remaining energy of the node decreases and the smaller the energy ratio is, the lower the probability of the node being selected as the cluster head node. If node *i* is the closest to the sink node, the distance factor is 1, otherwise, the distance factor is 0. Therefore, the range of the distance factor is between 0 and 1, and the closer the distance to the sink node, the closer the value to be 1.

#### Threshold Weight Selection

To ensure the rationality for the cluster head nodes selection, the optimal combined weighting (OCW) is used to adjust the three weights of the improved threshold, which combines the analytic hierarchy process (AHP) and the entropy method to ensure the objectivity of the threshold weight.

##### Weight of AHP

**Figure 2:**
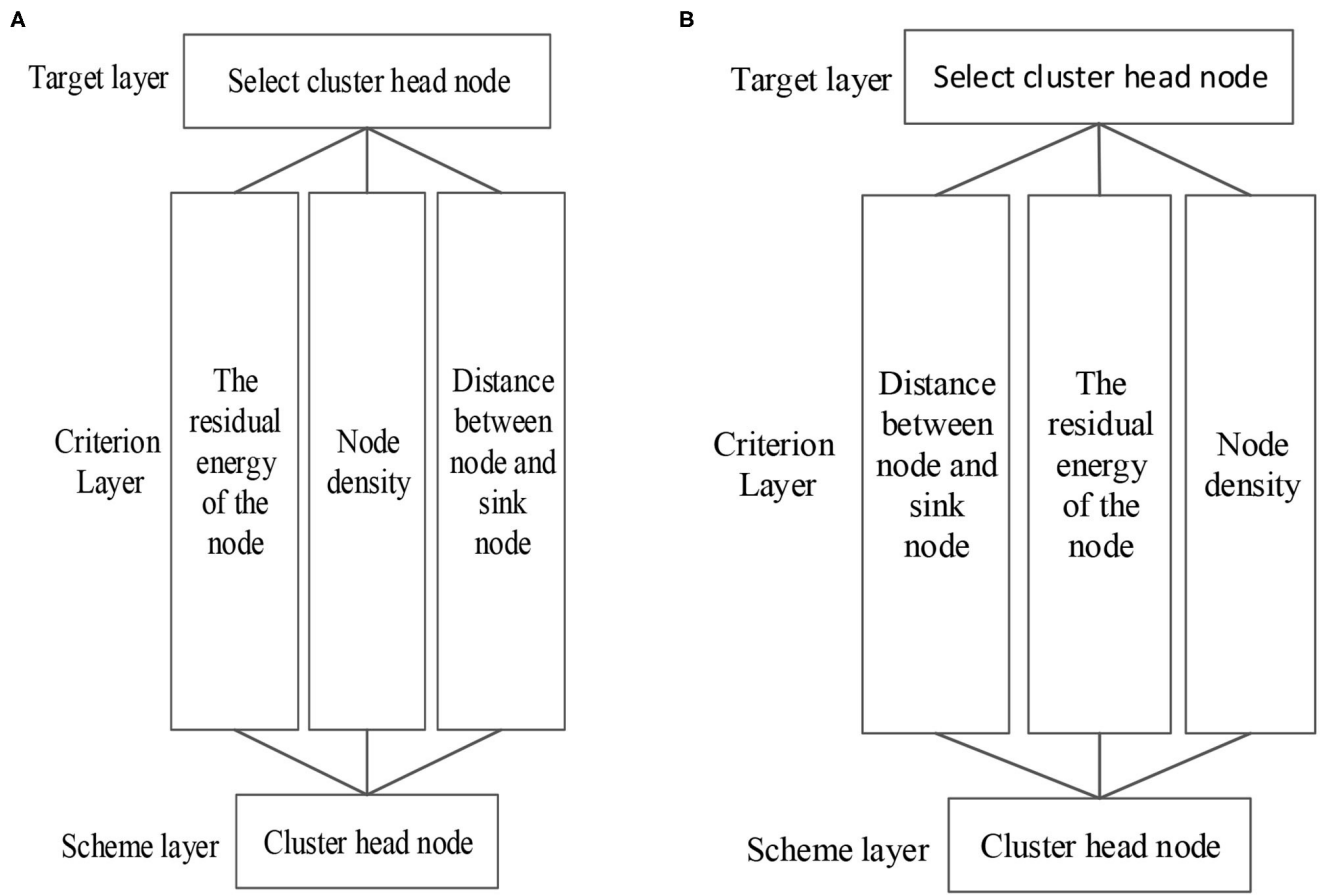
Hierarchical model of threshold weight.

The AHP method can decompose the problems into different levels with an evaluation index matrix to solve the maximum eigenvalues of the matrix and the corresponding eigenvector, so as to conduct a consistency test to obtain the weight of different evaluation indices (Yang et al., 2017). However, if the AHP method is used alone, the weight cannot be reasonable due to the large subjective component and certain persuasiveness. Still, the AHP method can be applied to determine the threshold weight in WSNs, and the steps are described as follows:

(1) Construction of the structural model of the cluster head node selection. The hierarchical model of the threshold weight in Region 1 (*d*_*i*_ ≤ *d*_0_) and Region 2 (*d*_*i*_ > *d*_0_) are depicted in Figures 2A,B.
(2) Determination of the evaluation index matrix. The residual energy, distribution density, and distance of the nodes are taken as evaluation indices, which are composed of the evaluation index matrix *C*, written as,
  (4) $$\begin{array}{l} C=\left[\begin{array}{ccc} 1 & c_{12} & c_{13}\\ c_{21} & 1 & c_{23}\\ c_{31} & c_{32} & 1 \end{array}\right] \end{array}$$
  where *c*_*ij*_ is the measurement value of the evaluation index, and $c_{ji}=\frac{1}{c_{ij}}$. The value of the *c*_*ij*_ usually adopts Santy's 1–9 scale method as listed in Table 2.
  According to the importance of the impact factors in different regions, evaluation index matrices corresponding to influencing factors can be obtained from different hierarchical models, as shown in Eq. (5).
  (5) $$\begin{array}{l} C=\left[\begin{array}{c} 1\;2\;5\\ \frac{1}{2}\;1\;3\\ \frac{1}{5}\;\frac{1}{3}\;1 \end{array}\right] \end{array}$$
(3) The calculation of the AHP weight. According to Eq. (6), the maximum eigenvalue and eigenvector of the evaluation index matrix can be obtained, λ_max_ and *W*, respectively, where the corresponding eigenvector is $W={\left[W_{1}\;W_{2}\;W_{3}\right]}^{T}$, and the obtained normalized weight is written in Eq. (7). The eigenvalue of Eq. (5) is 3.0037, and the weight of the energy, density, and distance are 0.581, 0.309, and 0.109 respectively.
  (6) $$\begin{array}{l} CW=\lambda_{\max} \end{array}$$
  (7) $$\omega_{Ai}=\frac{W_{i}}{\sum_{j}^{3}W_{j}}\;(i=1,2,3)$$
Consistency test. When the weights of the different impact factors are obtained, the final consistency test can be carried out to ensure the effectiveness of the obtained weights. The test expressions are expressed in Eqs. (8), (9), and (10). The consistency index of the weight values of the tested regions 1 and 2 is 0.0018, and the consistency ratio is 0.0032.
  (8) $$\begin{array}{l} CR=\frac{CI}{RI} \end{array}$$
  (9) $$\begin{array}{l} CI=\frac{\lambda_{\max}}{n-1} \end{array}$$
  (10) $$RI=\frac{\lambda_{\max}}{n-1}$$
  where, *RI* is the average consistency indicator, and *CR* is the consistency ratio. If *C* meets the consistency test, *CR* < 0.1. *CI* is a consistency indicator and *CI* = 0 with a high degree of consistency *C*.

**Table 2:** Evaluation index matrix value measurement table.

**Scale values**	***Meaning***
1	The two evaluation indicators are of the same importance
3	The previous evaluation index is more important
5	The previous evaluation index is important
7	The previous evaluation index is very important
9	The previous evaluation index is extremely important
2,4,6,8	The importance is the median value of the above adjacent indicators
Reciprocal	If the evaluation index i and j are measured as *c*_*ij*_,but $c_{ji}=\frac{1}{c_{ij}}$

##### Weight of Entropy Method

The entropy method is an objective weighting method without considering the correlation among factors. Here, the entropy method is applied to determine the threshold weight in WSNs.

Assuming WSNs containing *n* (*n* = 1,2,…, *N*) sensor nodes, the impact factor of the *n*th node is *f*_*nt*_ *t* (*t* = 1,2,…, *T*), to affect the threshold of the cluster head node selection.

(1) Analysis of the impact factors. According to Eq. (11), the impact factors are determined as the energy *f*_*n*1_, distance *f*_*n*2_, and relative density *f*_*n*3_ of the nth nodes,


(11) $$\begin{array}{l} f_{n1}=\frac{E_{i}}{E_{0}} \end{array}$$

(12) $$\begin{array}{l} f_{n2}=\frac{{dis}_{\max}}{{dmin}_{\max}} \end{array}$$

(13) $$\begin{array}{l} f_{n3}=\frac{l_{alive}}{N_{alive}} \end{array}$$

(2) The entropy method for the weight of impact factors determination.

(a) The above three impact indicators are normalized as,


(14) $$F_{nt}^{\prime }=\frac{f_{nt}-\min(f_{nt})}{\max(f_{nt})-\min(f_{nt})}$$

where max(*f*_*nt*_) and min(*f*_*nt*_) are the maximum and minimum of the impact factors, respectively. The evaluation matrix *R* of the normalized impact factor is obtained as,


(15) $$R=\left[\begin{array}{ccc} F_{11}^{\prime } & F_{12}^{\prime } & F_{13}^{\prime }\\ F_{21}^{\prime } & F_{22}^{\prime } & F_{23}^{\prime }\\ \cdots & \cdots & \cdots \\ F_{N1}^{\prime } & F_{N2}^{\prime } & F_{N3}^{\prime } \end{array}\right]$$

(b) The weight of the *t*th impact factor, *E*_*t*_ is expressed as,


(16) $$\begin{array}{l} E_{t}=\frac{1}{\ln N}\overset{N}{\underset{n=1}{\sum }}P_{nt}\ln P_{nt} \end{array}$$

(17) $$P_{nt}=\frac{F_{nt}^{\prime }}{\sum_{n=1}^{N}F_{nt}^{\prime }}$$

where *P*_*nt*_ is the proportion of the *t* impact factor of the *n*th node under all indicators of the node.

(c) The entropy value (weight) of each impact factor is written as,


(18) $$\begin{array}{l} \omega_{St}=\frac{1-E_{n}}{T-\sum_{t=l}^{T}E_{n}}\;0<\omega_{S}<1 \end{array}$$

##### Optimal Combined Weighting

The OCW is a method where both the quantitative and qualitative analyses are used to reasonably allocate the weight, and how to allocate the weight of the AHP and entropy is the key of this method (Yang et al., 2017). The weight calculation of the OCW method can be expressed as,


(19) $$\begin{array}{l} \omega_{t}=\lambda_{1}\omega_{Ai}+\lambda_{2}\omega_{St} \end{array}$$

where ω_*t*_, ω_*Ai*_, and ω_*St*_ are the weights of the optimal combination, AHP, and entropy method, respectively. λ_1_ and λ_2_ are the importance degrees of the AHP and entropy methods, respectively.

### A Bunch of Rules

Once the cluster head nodes are determined, they are broadcast inside the WSNs so that the rest sensor nodes are invited to join specific clusters. If a sensor node receives multiple invitation messages within a period of time, it will determine the distance between the cluster head nodes and itself according to the strength of the received information, then make the decision to join the closer cluster and transmit the requested information to the specific cluster head node. The cluster head node receives the distributed information from each member node and decides which nodes can be joined. Then, the clusters can be established, and it enters the data transmission stage.

## Energy Consumption Analysis of the Sensor Node

**Figure 3:**
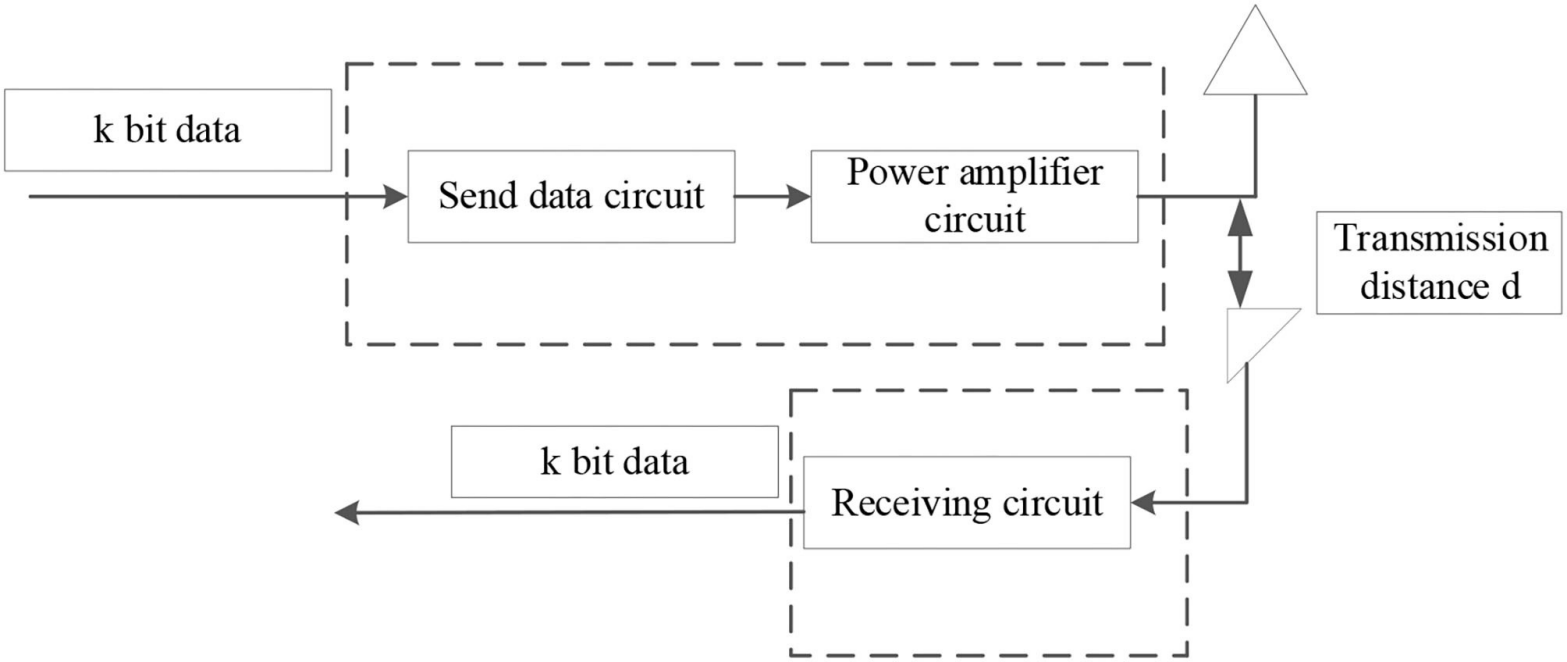
Wireless communication model of sensor nodes.

The differences in energy consumption with different routing protocols can be used to evaluate the transceiver characteristics of the sensor nodes. Hence, a typical radio energy consumption model is usually adopted, including the energy consumption of the data transmitting circuit and the power amplifier circuit (Yang et al., 2017), as demonstrated in Figure 3.

There are mainly two energy consumption models for the WSNs, i.e., free-space models and multipath fading models, the adoption of which depends on the distance between the sender and the receiver (Li et al., 2020) and written as,


(20) $$\begin{array}{l} E_{TX}\left(l,d\right)=E_{TX-elec}\left(l\right)+E_{TX-amp}\left(l,d\right)\\ \;=\left\{ \begin{array}{l} -l^{*}E_{elec}\;+l^{*}\varepsilon_{fs}*d^{2},\;if\;d\leq d_{0}\\ -l^{*}E_{elec}+l^{*}\varepsilon_{mp}*d^{4},\;if\;d>d_{0} \end{array}\right. \end{array}$$

where *E*_*TX*−*elec*_(*l*) is the loss of the transmitting circuit energy, *E*_*TX*−*amp*_(*l, d*) is the loss of the amplification circuit energy, and *E*_*elec*_ is the energy loss of the sender/receiver when performing data transmission. The calculation of the threshold of the data transmission distance is written as,


(21) $$\begin{array}{l} d_{0}=\sqrt{\frac{\varepsilon_{fs}}{\varepsilon_{mp}}} \end{array}$$

where ε_*fs*_ and ε_*mp*_ represent the amplification energy consumption parameters of the free-space model and the multipath fading model, respectively. The type of the transmitter amplifier determines the values of the two parameters.

## Optimization of the Transmission Path Between Clusters Based on IACO

### Flow Pattern Division

**Figure 4:**
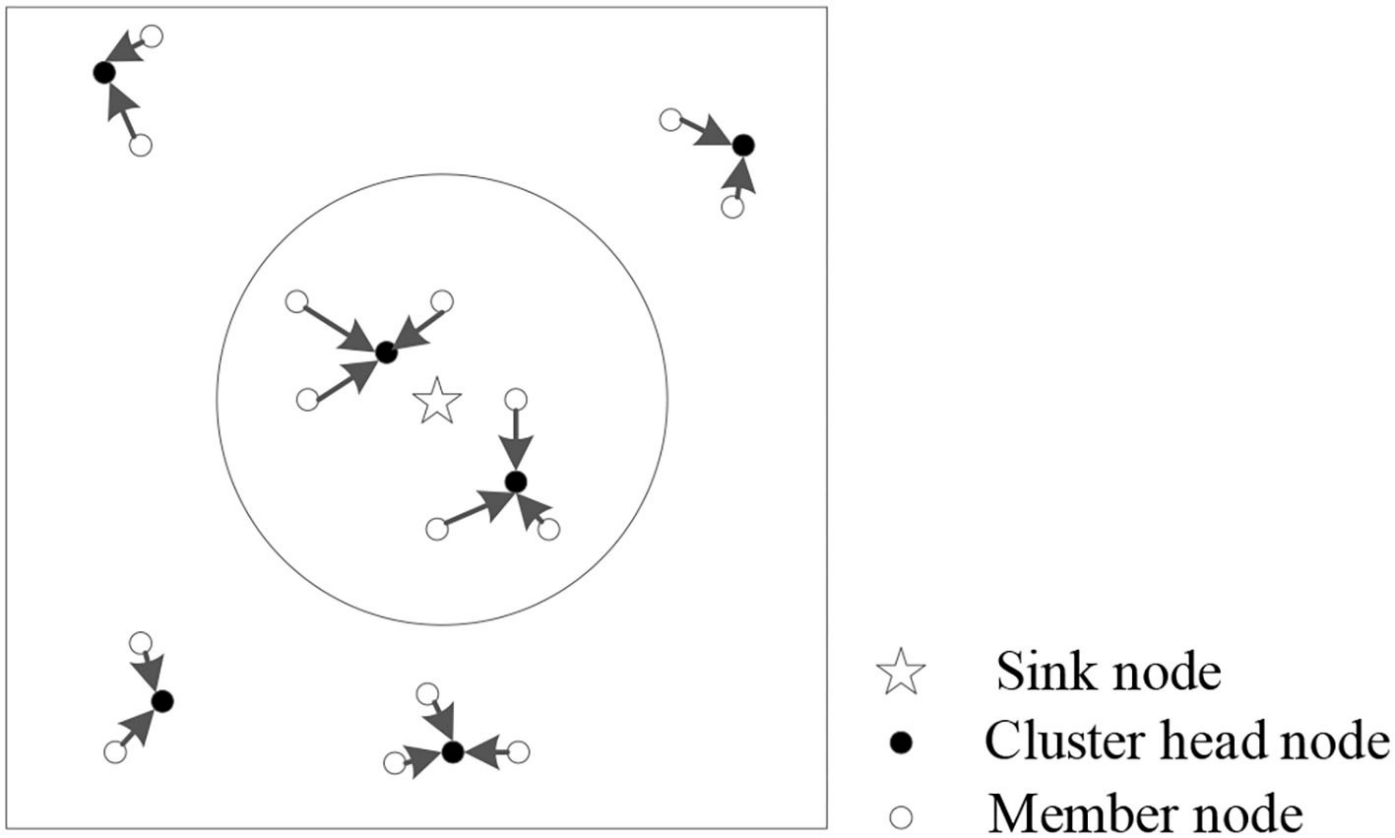
Schematic diagram of the zoning of wireless sensor network deployment node range.

According to the energy consumption model, the energy consumption of the nodes takes the distance threshold *d*_0_ as the intermediate value, so the regions can be divided by the data transmission distance threshold *d*_0_ in the LEACH protocol optimization. The range (*d*_min_ ≤ *d*_*i*_ ≤ *d*_0_) within the distance from the sink node *d*_0_ is area 1, and the range within the range of WSN nodes (*d*_0_ ≤ *d*_*i*_ ≤ *d*_max_) is area 2. The partitioning diagram is illustrated in Figure 4.

In WSNs, the node positions are usually fixed after deployment. When *d*_*i*_ ≤ *d*_0_, the distance between nodes will not be regarded as the main impact factor, so the weight in the threshold will change. In contrast, when *d*_0_ ≤ *d*_*i*_, the energy consumption of the node transmission and the distance between nodes increase to the fourth power. Hence, distance is the main impact factor. The weight in Eq. (19) will change with the importance of the impact factor.

### Transmission Path Optimization Among Clusters Based on the IACO Algorithm

The ACO algorithm is a heuristic optimization method, which can be used to search the optimal path through individual efforts and group cooperation *via* accumulated pheromones on the path with positive feedback (Song and Yao, 2017; Li et al., 2020). In WSNs, the optimization of the transmission data path of the routing protocol has the same characteristics as the ACO algorithm to obtain the best foraging route for ants. The application of the ACO algorithm in WSNs still has some limitations, including low path efficiency and the appearance of local optimal solution. Here, the developed IACO algorithm is applied to WSNs to conduct path optimization from three aspects, improved transition probability, path superiority, and pheromone updating mechanism.

#### Transfer Probability Improvement

The transition probability of the ACO algorithm considers only the path pheromone concentration and the distance between two nodes. The ant in the process of the optimal path search can also increase the probability of invalid path search and reduce the efficiency of path construction. Because the impact factors are not comprehensive, the flow of the data transmission of the nodes is larger, which could result in premature death affecting the network operation.

By using the ACO algorithm in WSN, the distance between two nodes is only considered in the next hop of the cluster node selection, which could cause more candidate nodes and a large amount of data transmission accompanied by more energy consumption. In this article, the transition probability is fully considered with the node energy for the ants' node search ability enhancement, so as to speed up the convergence speed, and the developed transition probability function is formatted as,


(22) Pijk(t)={(τij)α(ηij)β(Eja)γ∑s∈allowedk(τis)α(ηjs)β(Eja)γ ,   s∈allowedk0                          ,      s∈allowedk

(23) $$\begin{array}{l} E_{ja}=\frac{E_{j}}{E_{iave}} \end{array}$$

where *allowed*_*k*_ is the cluster head node set where *k* nodes have not been reached, *E*_*j*_ is the remaining energy of the next hop node, and *E*_*iave*_ is the average energy of the adjacent nodes of node *i*. τ_*ij*_ is the pheromone concentration of the path from node *i* to node *j*, and η_*ij*_ is the heuristic function to be defined for the cluster head node

Based on the forwarding distance *j* node and the distance of the aggregation node, written as,


(24) $$\begin{array}{l} \eta_{ij}=\frac{1}{d_{ij}+d_{js}} \end{array}$$

where *d*_*ij*_ is the Euclidean distance between node *i* and the next hop node *j*, and *d*_*js*_ is the Euclidean distance between the next hop node *j* and the sink node.

#### Path Superiority Determination

When all ants are transferred to the sink node, each ant corresponds to a transmission path, so the path superiority degree can be used as the standard to measure the optimal path. The ant with a higher superiority degree is the optimal transmission path. In the previous path optimization process, the path with higher average energy is highly likely to be the best transmission path; however, the mean energy cannot represent the current node energy level. For instance, if certain nodes have high energy mean, but the energy difference between the actual nodes is substantially large, it will cause premature death of the nodes and failure of the transmission path. The path superiority is thus reflected from the lower hop count, higher mean energy, and uniform energy distribution. The higher the path superiority, the higher quality of the selected transmission path.

The variation coefficient is defined by the ratio between the SD and mean of the data, which is used to evaluate the difference degree of the data distribution. The smaller the coefficient of the variation, the more uniform the data distribution, and the smaller difference between the data (Zhu, 2017). Therefore, the path variation coefficient *P*_*cv*_ is used to analyze the energy balance of the nodes.


(25) $$\begin{array}{l} P_{cv}=\frac{P_{sd}}{P_{m}}=\frac{\sqrt{\frac{1}{\delta }\sum_{i=1}^{\delta }{\left(E\left(i\right)-E_{ave}\right)}^{2}}}{E_{ave}} \end{array}$$

where *P*_*sd*_ is the SD of the energy of all nodes in the path *P*_*m*_, *E*_*ave*_ is the mean energy of all nodes in the path, and δ is the number of nodes contained in the path.

Hence, the path superiority *P*_*s*_ can be written as,


(26) $$\begin{array}{l} P_{s}=\frac{E_{\min}}{E_{con}\frac{1}{P_{cv}}+\frac{1}{J}} \end{array}$$

where *E*_min_ is the minimum of the node energy, *E*_*con*_ is the sum of the node energy consumption, and *J* is the total hop number in the path.

#### Pheromone Updating

In the ACO algorithm, the pheromone concentration varies according to the length of the path, and ants plan the next route according to the pheromone concentration of different paths. Ants tend to choose paths with higher pheromone concentrations, and other paths are ignored. As a result, the path searching will fall into the local optimization, the ant will no longer search for new paths and the path searching process stops. Therefore, the IACO algorithm adds local and global pheromone updates to inter-cluster transmission path planning to avoid such local optimum problems. The developed pheromone updating mechanism is written as,


(27) $$\begin{array}{l} \tau_{ij}\left(t+1\right)=\left(1-\rho \right)\tau_{ij}\left(t\right)+\rho \Delta \tau_{ij}\left(t\right) \end{array}$$

(28) $$\begin{array}{l} \Delta \tau_{ij}\left(t\right)=\overset{N}{\underset{k=1}{\sum }}\Delta \tau_{ij}^{k}\left(t\right) \end{array}$$

where ρ is the parameter regulating the pheromone volatilization speed, Δτ_*ij*_(*t*) is the pheromone increment in the path, Δτ_*ij*_(0) = 0 at the initial time, and $\Delta \tau_{ij}^{k}\left(t\right)$ is the pheromone concentration left by the *k*th ant in the path (i, j).

- Local pheromone update: If the ant node carries out data forwarding from the cluster head node *i* → *j*, the pheromone concentrations of the corresponding paths should be locally updated,

(29) $$\Delta \tau_{ij}^{k}(t)=\frac{E_{r}(j)}{d(i,j)}$$

where *E*_*r*_(*j*) is the number of the member nodes in the next hop node.

- Global pheromone update: When all ants move to the sink node, each ant corresponds to a transmission path, and the pheromone concentration of the path is updated globally. Based on the path superiority, the global update rules is written as,

(30) $$\begin{array}{l} \Delta \tau_{ij}^{k}(t)=\frac{1}{P_{s}} \end{array}$$

**Figure 5:**
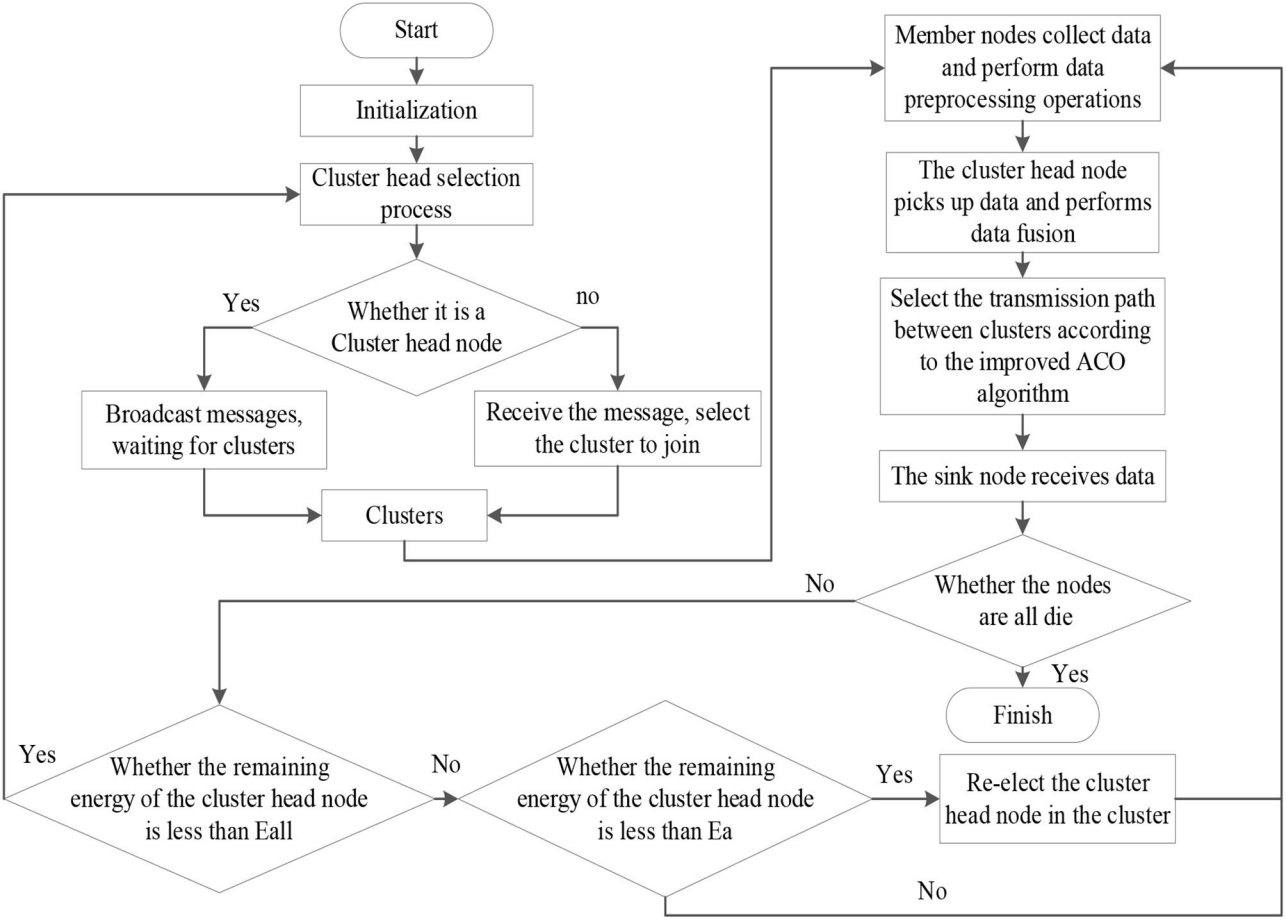
Improved LEACH protocol flowchart.

The LEACH protocol is optimized with the improved clustering algorithm to cluster sensor nodes, and the influence factors of nodes in different network regions are different so as to replace the cluster head nodes dynamically. When the energy of the cluster head node reaches the limit value, the cluster is updated and the data are fused in the cluster head node. Although the IACO algorithm is used to find the optimal transmission path between clusters, it can effectively avoid the stagnation of the IACO algorithm in the local optimum. The specific process is depicted in Figure 5.

## Experimental Verification and Analysis

### Experimental Environment Setting

**Figure 6:**
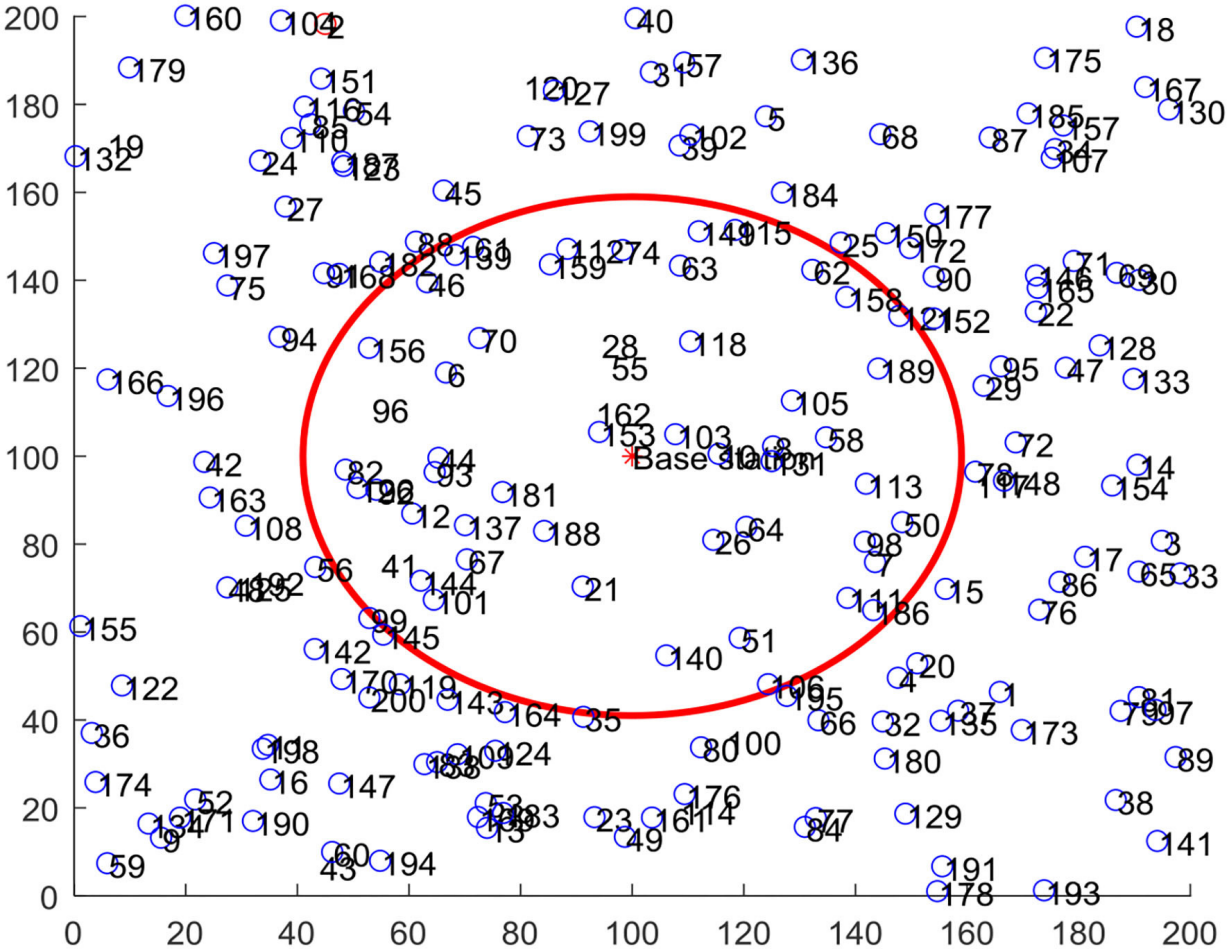
Sensor node deployment diagram.

The experiment verification is carried out in a MATLAB simulation environment, and 200 sensor nodes are deployed in a 200 × 200 m network area. The node deployment within the network is shown in Figure 6, where the circle with sink node is the center, the area with the threshold distance *R* as the radius of the circle is area 1, and the rest is area 2. The parameters of the basic network and IACO algorithm in the experiments are listed in Tables 3, 4.

**Table 3:** Basic parameter settings of the network.

**Parameter**	**Parameter value**
Network area	200 × 200 m
Number of nodes	200 individuals
The location of the sink node	(100, 100)
Node's initial energy *E*_0_	0.2J
Energy consumption at the receiving end *E*_*RX*_	50 nJ/bit*m^2^
Energy consumption of the sender *E*_*TX*_	50 nJ/bit
Energy consumption of data fusion *E*_*DA*_	5 nJ/bit
Free space model energy consumption parameters ε_*fs*_	10 pJ/bit*m^2^
Amplified energy consumption parameters of the multipath model ε_*mp*_	0.0013 pJ/bit*m^2^
Control packet size	100 bits
Packet size	3,000 bits

**Table 4:** Basic parameter setting of IACO algorithm.

**Parameter**	**Parameter value**
Pheromone concentration weight factor α	1
Heuristic function weight factor β	5
Node energy consumption weight factor λ	4
Pheromone Volatilization Coefficient ρ	0.1

### Performance Indicators

The life cycle of the network, the total energy consumption of the network, and the data received by the sink node are taken as indicators to evaluate the quality of the routing protocol. The specific analysis is as follows:

1. Network life cycle: the duration from the normal operation of the WSN after the successful layout to the death of the last node. Three indicators were selected for evaluation, namely, the number of rounds in which the first node died (indicator 1), the number of rounds in which 10% of nodes died (indicator 2), and the number of rounds in which all nodes died (indicator 3). In most cases, the appearance of the dead nodes leads to the deterioration of the network detection quality, so the number of rounds where the first node dies is of particular importance.
2. Total network energy consumption: the total energy consumption of all nodes in the network during implementation. This index can reflect the balance degree of energy consumption. In the simulation, the size of the packet and control packet is set, and the energy consumptions of different nodes are calculated through the network operation.
3. Data received by the sink node: data received by the sink node after each round of the network operation.

### Experimental Results and Analysis

The simulation results of different routing protocols are analyzed based on the above performance indicators to verify the effectiveness of the ILEACH protocol.

**Figure 7:**
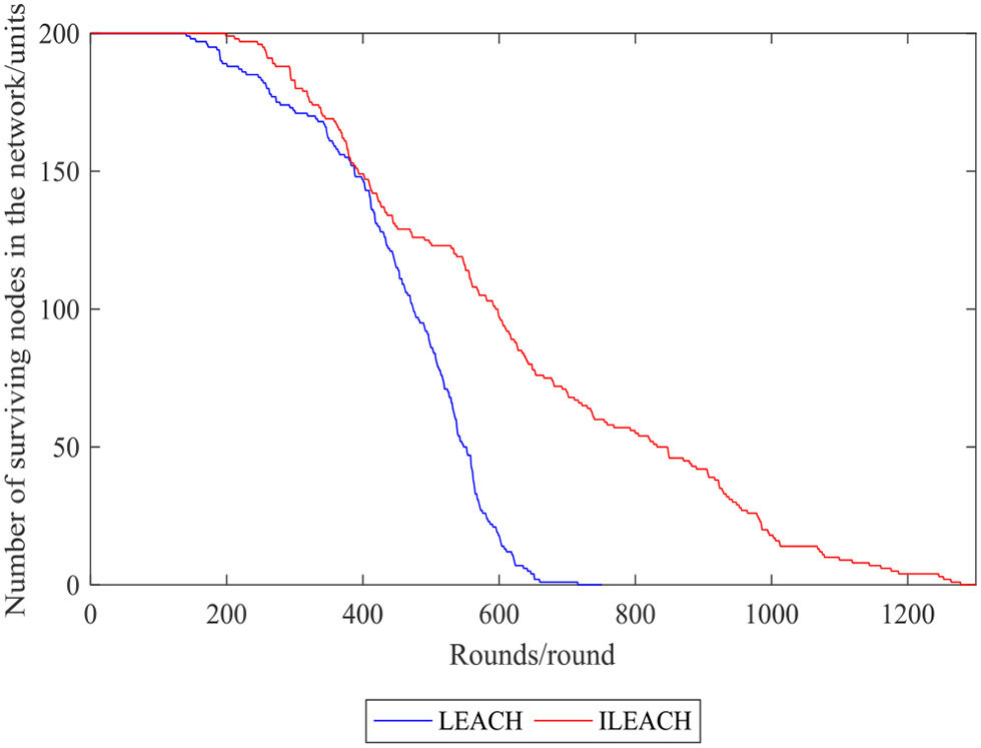
Comparison curve of the number of surviving nodes in the network.

**Figure 8:**
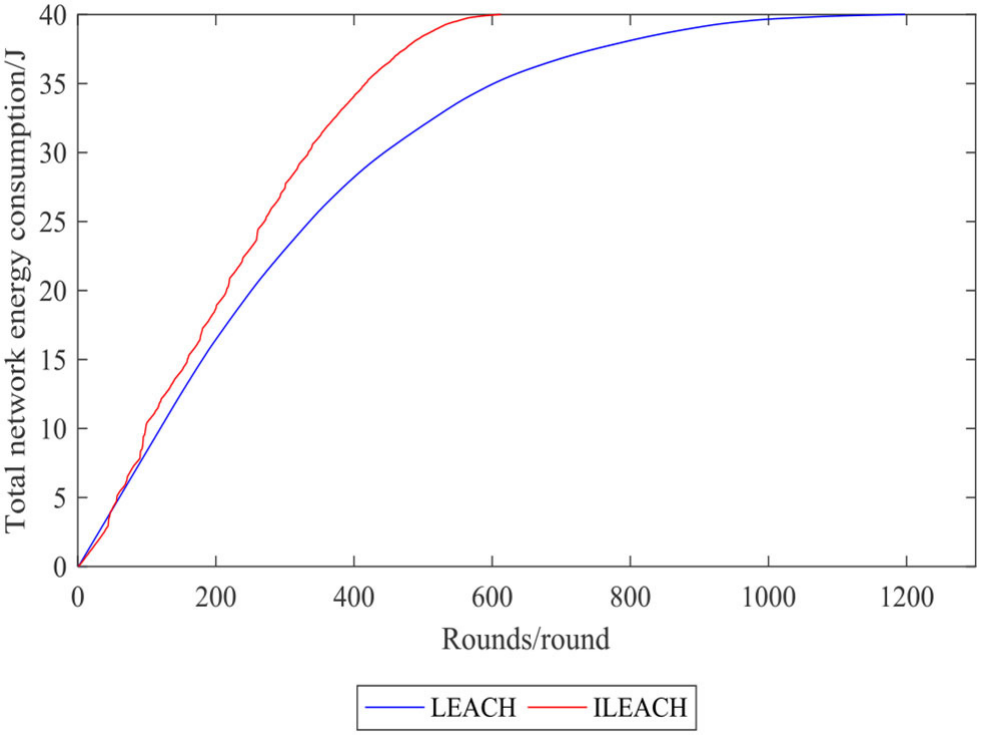
Comparison curve of total network energy consumption.

Figures 7, 8 compare the number of surviving nodes and total network energy consumption of the LEACH protocol and the ILEACH protocol, respectively. To be specific, the impact of the ILEACH protocol on the network life cycle is analyzed *via* indicators 1 and 2, as shown in Figure 8.

**Figure 9:**
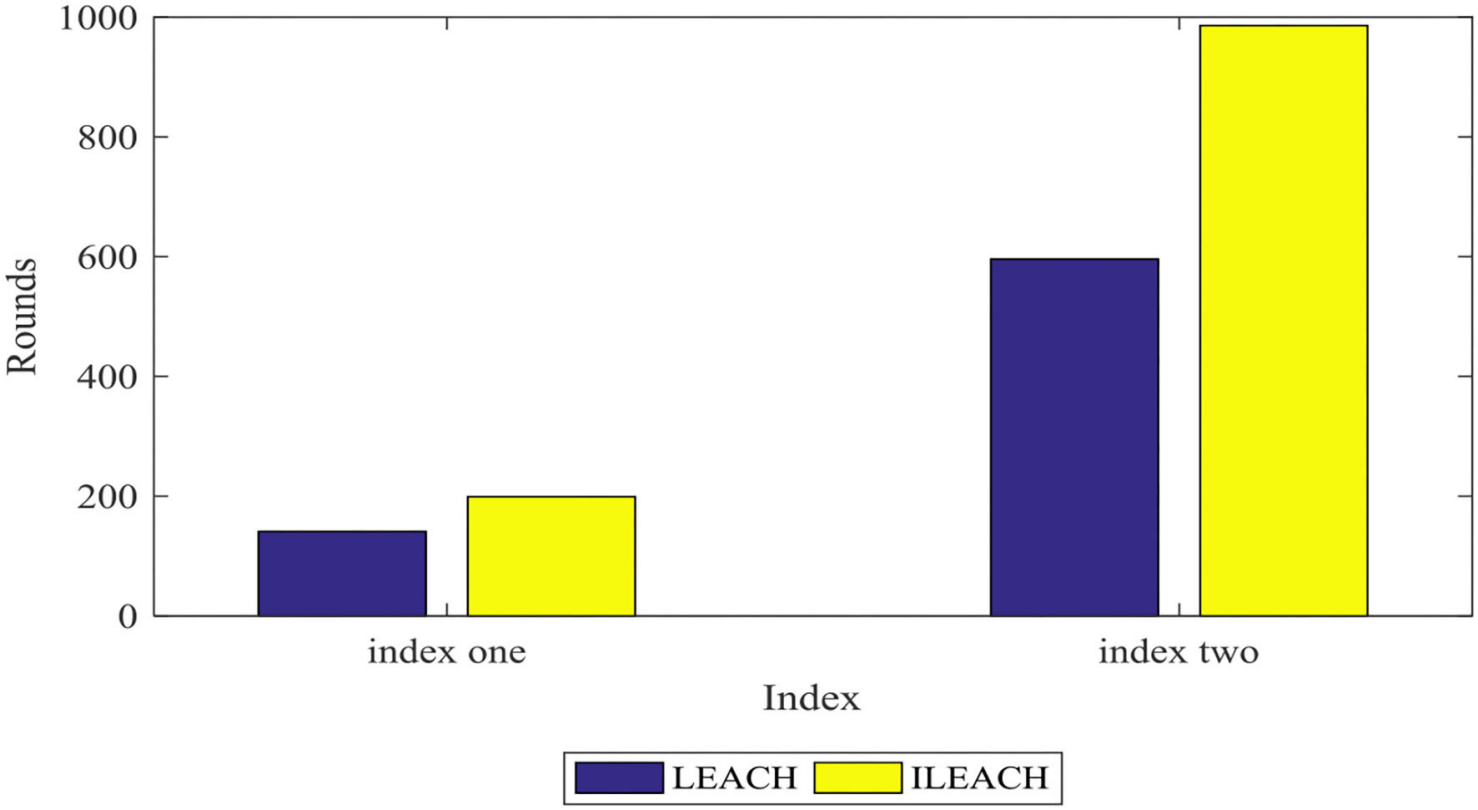
Indicator analysis histogram.

Figure 9 indicates that indicator 1 of the LEACH protocol and the ILEACH protocol appears in rounds 141 and 199, respectively, and indicator 2 appears when the network operates to rounds 596 and 986. When the LEACH protocol reaches ~600 rounds, most nodes have no power, but the ILEACH protocol can be extended to ~1,200 rounds. The network needs to be initialized in the early stage, which consumes energy quickly, and there are fewer surviving nodes in the later stage. However, ILEACH can effectively balance the energy consumption of nodes by considering the energy consumption of nodes in the process of clustering and data transmission, and the network energy consumption varies slowly in the later period. It is proven that the improved threshold and the replacement mechanism of the cluster head nodes can reduce the energy consumption of the nodes, and the ILEACH protocol can prolong the network life cycle much longer compared with the LEACH protocol.

**Figure 10:**
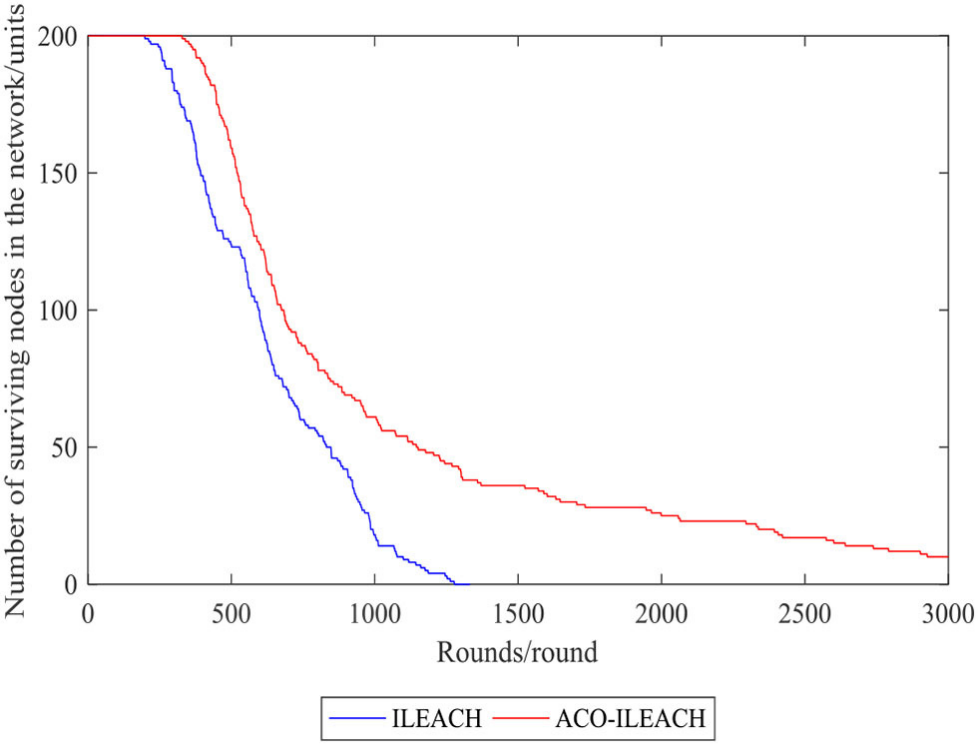
Comparison curve of the number of surviving nodes in the network.

**Figure 11:**
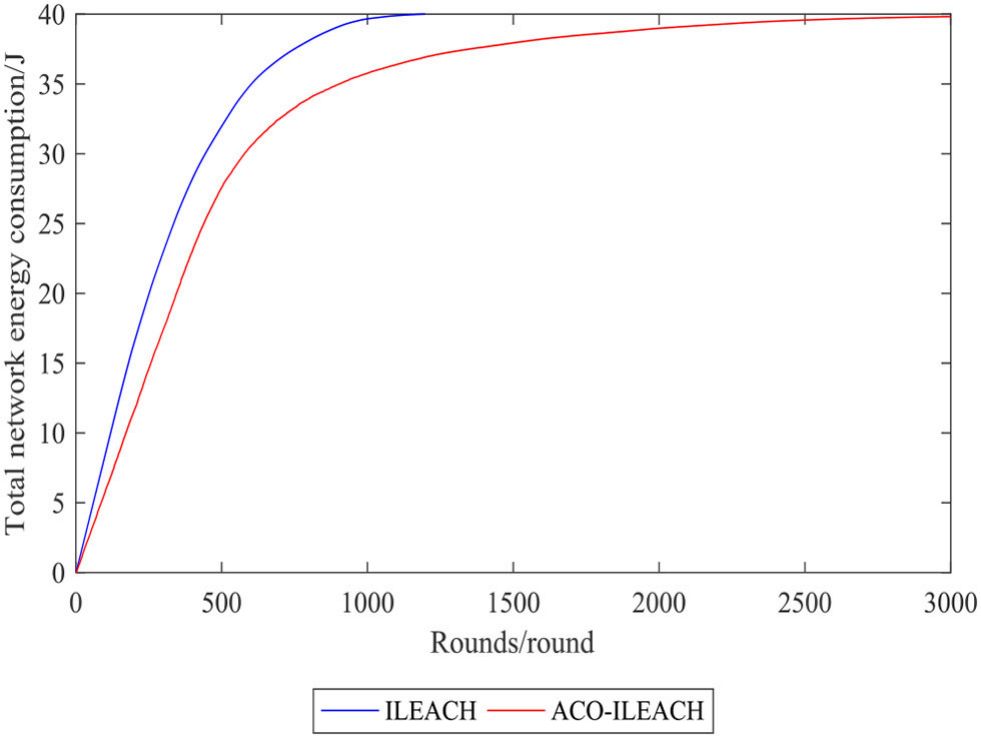
Comparison curve of totalnetwork energy consumption.

**Figure 12:**
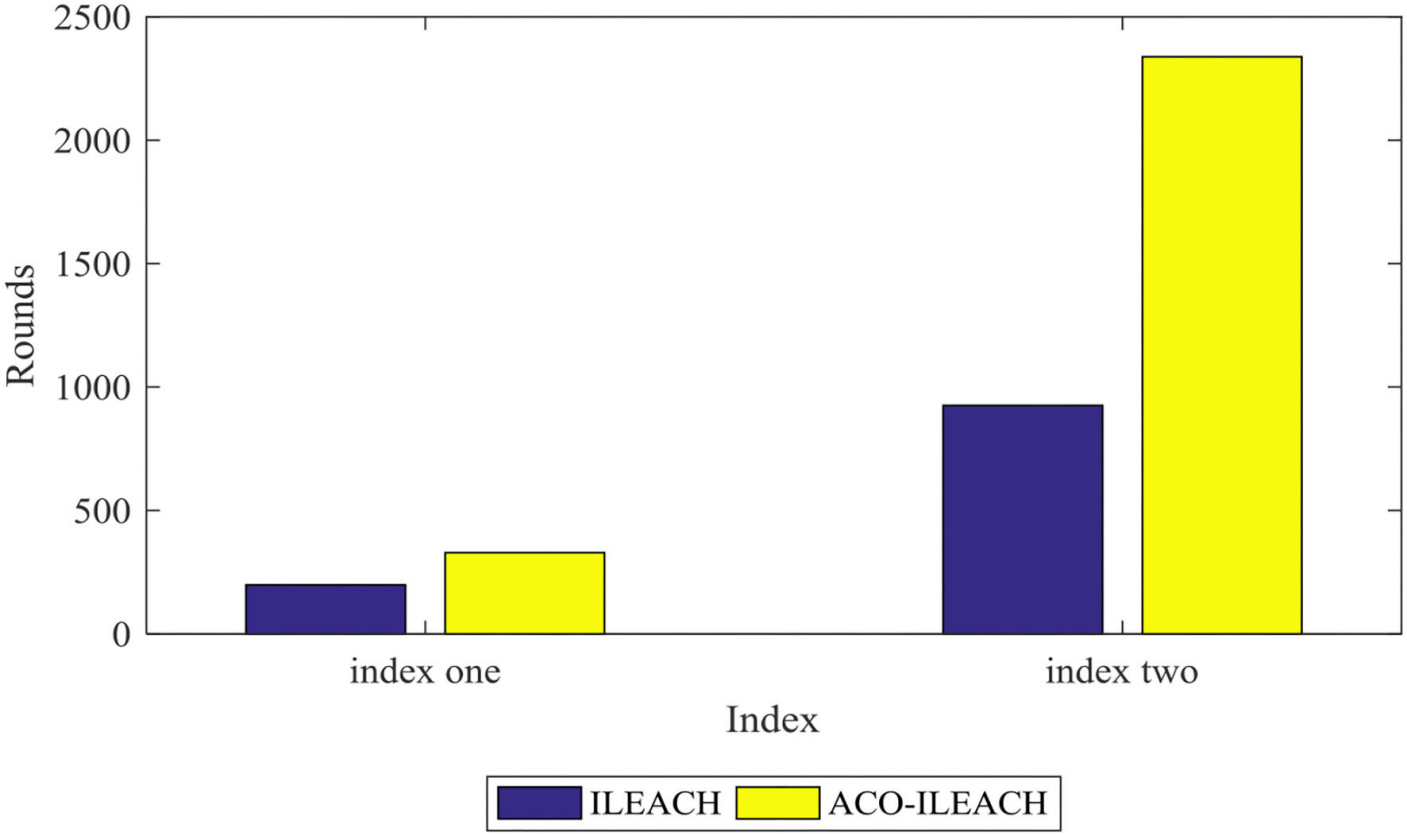
Indicator analysis histogram.

Figures 10, 11 compare the number of the surviving nodes and total network energy consumption between the LEACH of ACO (ACO-LEACH) protocol and the LEACH of IACO (IACO-LEACH) protocol, where Figure 11 is the histogram of the index. Figure 12 illustrates that indicator 1 of the ACO-LEACH protocol and IACO-LEACH protocol occurs in rounds 199 and 329, respectively, and indicator 2 occurs in rounds 986 and 2,338. Table 5 displays the number of the first node deaths, 10% node deaths, and all node deaths under different protocols. In conclusion, the developed IACO algorithm can gradually find the optimal transmission path for the WSN operation and save energy in the data transmission stage effectively.

**Table 5:** Comparison table of the network life cycle of different protocols.

**Protocol**	**Number of rounds in which the first node died**	**Number of rounds in which 10% of the nodes died**	**Number of rounds in which all nodes died**
LEACH	141	596	659
ILEACH	199	986	1,296
ACO-ILEACH	329	2,338	3,487

## Conclusion

An algorithm based on OCW and IACO is proposed in the article to solve the problem of high energy consumption of the traditional LEACH protocol in WSNs. The ILEACH protocol adopts the cluster head node replacement mechanism to reduce the energy consumption considering the energy, density, and nodes distance for the threshold selection, which can effectively avoid the randomness of the clustering. Furthermore, the OCW is used to dynamically change the weight of the nodes according to the different impact factors of nodes in different regions. The developed IACO algorithm can optimize the transfer probability of the sensor nodes with the local update and global update strategies, which can prolong the life cycle of the network to a certain extent. The network environment is deployed through MATLAB simulation software to verify the feasibility of the ILEACH protocol.

## Data Availability Statement

The original contributions presented in the study are included in the article/supplementary material, further inquiries can be directed to the corresponding author.

## Author Contributions

XC and JZ proposed the idea in this study. CX and XL designed the experiment, performed the simulation experiments, analyzed the experiment results, and wrote the manuscript. JL corrected the manuscript. All authors contributed to the article and approved the submitted version.

## Funding

The project (work) was supported by the National Natural Science Foundation of China Program (No. 62073198) and the Major Research Development Program of Shandong province of China (No. 2016GSF117009).

## Conflict of Interest

XL is employed by Shandong Senter Electronic Co., Ltd. The remaining authors declare that the research was conducted in the absence of any commercial or financial relationships that could be construed as a potential conflict of interest.

## Publisher's Note

All claims expressed in this article are solely those of the authors and do not necessarily represent those of their affiliated organizations, or those of the publisher, the editors and the reviewers. Any product that may be evaluated in this article, or claim that may be made by its manufacturer, is not guaranteed or endorsed by the publisher.
